# Prevalence of Stroke in Individuals with Sickle Cell Disease Pre- and during Hydroxyurea Uses: A Descriptive Cross-Sectional Study in Tanzania

**DOI:** 10.1155/2024/7950925

**Published:** 2024-03-19

**Authors:** Belinda Nestory Moshi, Erick G. Philipo, Nancy F. Kileo, Joseph Matobo, Emili Yondu, Dionis Ikunda, Daniel Kandonga, Koga M. Luhulla, Manase Kilonzi

**Affiliations:** ^1^School of Pharmacy, The Muhimbili University of Health and Allied Sciences, P.O. Box 65013, Dar es Salaam, Tanzania; ^2^School of Diagnostic Medicine, The Muhimbili University of Health and Allied Sciences, P.O. Box 65001, Dar es Salaam, Tanzania; ^3^Department of Pediatrics, The Muhimbili National Hospital, P.O. Box 65000, Dar es Salaam, Tanzania; ^4^School of Medicine and Dentistry, University of Dodoma, P. O. Box 395, Dodoma, Tanzania; ^5^Sickle Cell Programme, Muhimbili University of Health and Allied Sciences, P.O. Box 65001, Dar es Salaam, Tanzania

## Abstract

Sickle cell disease (SCD) is an inherited blood disorder that leads to a variety of complications, including stroke. The use of hydroxyurea (HU) is reported to lessen the frequency and burden of stroke in SCD patients. However, less is known about the prevalence of stroke in SCD patients pre- and during the use of HU in sub-Saharan African (SSA) countries. Therefore, the study assessed stroke prevalence before and during uses of hydroxyurea among SCD patients in Tanzania. A hospital-based descriptive cross-sectional study was conducted at the sickle cell clinics in Dar es Salaam, Tanzania, from April 2023 to May 2023. A total of 228 participants were recruited, and data on demographic and clinical characteristics, HU use, and history of stroke were collected using a checklist from the respective patients' medical records and verbal communication with the patients or caregivers. Data analysis was done using SPSS software version 25, and findings are summarized using frequency and percentages. Out of 228 enrolled SCD patients, 124 (54.4%) were females, 109 (47.8%) were aged between 6 and 12 years, 226 (99.1%) were not married, 181 (79.4%) had primary education, and 209 (95%) were unemployed. The prevalence of stroke pre-HU use was 28 (12.3%) and 6 (2.6%) after starting using HU. Out of 6 with stroke after starting using HU, 3 (50%) had a history of stroke pre-HU uses. The study showed that the prevalence of stroke among SCD patients is significantly reduced after HU use. The findings suggest the need for stakeholders to implement measures to ensure eligible SCD patients are kept on HU.

## 1. Introduction

Sickle cell disease (SCD) is a global burden, which is estimated to affect 14 million newborns by 2050 [1]. SCD is a group of autosomal recessive hemoglobinopathies that occur due to a genetic mutation of hemoglobin, whereby glutamic acid is substituted by valine in position 6 of the beta-globin chain leading to an abnormal hemoglobin known as hemoglobin S (HbS) [2]. Compared to normal hemoglobin, HbS has low solubility, and with low oxygenation, it results in the polymerization and formation of rigid sickle-shaped red blood cells. Polymerization of the HbS molecules by deoxygenation is the main hallmark for most complications and end-organ damage, however, low pH, high temperature, and concentration of HbS in the erythrocytes surplus the risk [3, 4].

Stroke is one of the significant causes of morbidity and mortality in individuals with SCD [5]. World Health Organization (WHO) describes stroke as a clinical syndrome with a series of neurological events that occur when there is a disruption or blockage of the blood flow to the brain, with clinical signs of focal or global disturbance of cerebral function, lasting for more than 24 hrs or leading to brain tissue damage, potential long-term disabilities, and even death [6]. In SCD patients, a stroke can be an overt stroke or silent cerebral infarction. Overt stroke refers to a symptomatic event characterized by the sudden onset of neurological deficits. Silent cerebral infarctions, on the other hand, are asymptomatic and typically discovered incidentally through brain imaging [5].

Children with SCD are particularly susceptible to strokes, with the highest risk occurring between the ages of 2 and 5 years, while the majority succumb to more complications [7]. To prevent these complications, treatment of SCD early in life is important, and stem cell transplant is a curative therapy [8]. However, due to the costs, mismatching, and anticipated posthematopoietic stem cell transplantation (HSCT) complications, disease-modifying agents such as voxelotor and hydroxyurea (HU) are mostly used globally [9, 10]. HU is a potent disease-modifying therapy for SCD which is found to be safe and feasible for low-middle-income countries (LMICs) [11].

HU, also known as hydroxycarbamide, is an oral drug used as a disease-modifying treatment for various hematological disorders, one of them being sickle cell anemia (SSA). It is a cytotoxic agent that exerts its effects through multiple mechanisms, primarily by influencing erythropoiesis and F-cell production, which in turn determines HbF level and hemolysis and hence increases the production of fetal hemoglobin (HbF), which helps to prevent the sickling of RBCs [12]. It leads to the improvement of RBC deformability and reduces chronic inflammation which in turn decreases hemolysis in patients and decreases the incidence of acute painful vaso-occlusive events, infections, transfusions, hospitalizations, and even death [11]. Furthermore, it reduces the incidence and/or recurrence of strokes and improves overall neurological outcomes.

WHO recommends all individuals with SCD in LMICs to be placed on HU for their entire life [13]. In Tanzania, based on the SCD database owned by the Sickle Pan-African Research Consortium (SPARCO)-Sickle Cell Center under the Department of Hematology and Blood Transfusion at the Muhimbili University of Health and Allied Sciences (MUHAS), over 30% of the patients are on HU. Despite increases in the number of SCD on HU in Tanzania, few have been documented on the occurrence and prevalence of stroke. Therefore, this study aimed to assess the prevalence of stroke among SCD patients' pre- and post-HU use in Dar es Salaam, Tanzania.

## 2. Methods

### 2.1. Study Design and Setting

A hospital-based descriptive cross-sectional study was conducted from April 2023 to June 2023 among patients with SCD and parents/caregivers residing in the Dar es Salaam region, Tanzania. Dar es Salaam contains five sickle cell clinics, located in the national and regional referral hospitals (RRHs) (Muhimbili, Temeke, Mwananyamala, and Amana). Only three out of five sickle cell clinics were selected (Muhimbili National Hospital, Temeke, and Amana RRHs), based on the criteria that these clinics were dispensing HU. The clinics are under the supervision of the MUHAS-SPARCO sickle cell center. During the clinic days, medical doctors from the MUHAS-SPARCO sickle cell center oversee clinics in all hospitals. In total, Dar es Salaam is estimated to have around 3000 SCD patients, and the majority attend clinics in the abovementioned five hospitals.

### 2.2. Study Population and Eligibility Criteria

The study involved all SCD patients on HU for more than three months. Written informed consent was obtained from the patient or parent/caregiver before enrolling in the study.

#### 2.2.1. Hydroxyurea Therapy in Tanzania

According to Tanzania' sickle cell disease clinical management guidelines, adults with SCD (≥18 years) should start with 15 mg/kg/day and adjustment to 5–10 mg/kg/day should be considered for individuals with chronic kidney disease. Those below <18 years of age are given 20 mg/kg/day, and the guideline insists that the drug should be given from 9 months of age. Since the most available HU formulation in Tanzania contains 500 mg, the guideline advises the dosage to be calculated per week and the provider to see how the patient can take the drug to avoid toxicity. Before initiation of HU, some hematological, kidney, and liver function tests include hemoglobin, platelets, absolute neutrophil count, bilirubin total and direct, serum creatinine, and serum ALT. The primary target is to achieve a hemoglobin level of 10 g/dl; therefore, an assessment is performed every 8 weeks, and 5 mg/kg/day is added. Escalation of the dose will stop when 10 g/dl is reached or the maximum tolerable dose of HU which is 35 mg/kg/day is attained. In case the patient attains 10 g/dl before reaching 35 mg/kg/day and maintains the level over 12 weeks or more, the dose is marked as a therapeutic dose (TD) and will be maintained while monitoring the Hb level [14, 15].

### 2.3. Sample Size Calculation, Sample Size, and Sampling Technique

A total of 228 SCD patients were recruited. The estimated sample size was calculated from the Kish and Leslie formula for a cross-sectional study *z*^2^ × *p* (1−*p*)/*d*^2^ (1965). Assuming a prevalence of stroke (*p*=16.9%) obtained from a similar study conducted in Northwest Tanzania (30), with a precision of *d* = 5% and a confidence level of 95%, a minimum of 216 SCD patients was obtained. The participants were recruited consecutively.

### 2.4. Data Collection Procedure

Data were collected using a data abstraction tool (checklist). The data abstraction tool contains a section for documenting sociodemographic characteristics (such as age, gender, and marital status) and a section for stroke episodes pre- and during HU use. Data were collected by one of the investigators (BNM) who were fully involved from the conceptualization of the study to proposal development in collaboration with the respective clinic medical doctor. The process of data collection involved reviewing the medical records of patients and interviewing the patient or parent/caregiver in case of missed information or clarification.

### 2.5. Data Analysis

Data from the data abstraction tool were entered into an MS Excel sheet and transferred to the SPSS software version 26 for analysis. Findings are summarized using frequency and percentages.

## 3. Results

### 3.1. Demographic Characteristics of Study Participants

A total of 228 participants were interviewed, of which 124 (53.1%) were female and 109 (45.6%) participants were aged between 6 and 12 years. The majority (172 (75.4%)) of participants were diagnosed with SCD at the age of 0–5 years. Besides, 181 (979.4%) of the participants had primary education, and the majority (209 (95.0%)) were unemployed (Table 1).

### 3.2. Prevalence of Stroke Pre- and During HU Uses among Participants

**Figure 1:**
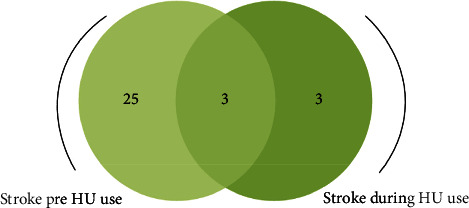
The number of study participants with stroke pre- and during HU use (*n* = 28).

Out of the 228 participants, the majority (198 (86.8%)) have been on HU for more than 6 months and more than half (132 (57.9%)) were using 3500 mg of HU per week. The prevalence of stroke pre-HU use was found to be 28 (12.3%) and 6 (2.6%) after starting using HU (Table 2). Out of 6 with stroke after starting using HU, 3 (50%) had a history of stroke pre-HU uses (Figure 1).

### 3.3. Demographic Characteristics and Stroke Status among Study Participants

Among the three age groups, the 6–12 years age group had a remarkable decrease in stroke prevalence, from 11.9% pre-HU use to 0.9% post-HU use. In the group with >12 years, there was stroke recurrence post-HU use with a prevalence of 3 (4.8%). Among females, stroke prevalence decreased from 14 (11.3%) pre-HU use to 3 (2.4%) during HU use. For males, it reduced from 14 (13.5%) pre-HU use to 3 (2.9%) during HU use. Stroke recurrence is seen to be greater in males (2 (1.9%)) than in females (1 (0.8%)). Education wise, there was a high stroke prevalence in individuals with a primary education in both pre-HU use (23 (12.3%)) and during HU use (5 (2.8%)), with a recurrence of 3 (1.7%). Among the unemployed participants, stroke prevalence reduced from 23 (11.0%) to 5 (2.4%) during HU use (Table 3).

## 4. Discussion

This study was conducted to assess the prevalence of stroke pre- and during HU use among SCD patients in Dar es Salaam, Tanzania. The study found that the prevalence of stroke pre-HU use was 12.3% and 2.6% after starting using HU. Out of the 6 SCD patients with stroke after starting using HU, 3 (50%) had a history of stroke pre-HU uses. The study also observed that the majority (75%) of the participants were on HU, taking a dose of >3500 mg/week for more than 6 months. The study further found that pre-HU stroke is significantly prevalent in SCD patients aged 6–12 years.

Similar to previous studies which report that the prevalence of stroke among SCD ranges between 2.90% and 16.9% [16], our study found the prevalence of stroke to be 12.3% pre-HU uses. The results signify that stroke is among the major complications of SCD as the studies involved diverse age groups [16, 17]. The present study reported an almost 79% reduction in the prevalence of stroke among SCD patients after starting using HU, i.e., from 12.3% to 2.6%. The findings are consistent with what was reported in Nigeria (2019), in which a study reported the reduction of stroke recurrence in SCD patients from 14 cases of pre-HU use to 5 cases of strokes post-HU use [18]. HU is reported to increase the fetal hemoglobin (HbF) level and myelosuppression which results in the lowering of leucocytes, decreasing sickling, and improving the rheological and flowability properties of erythrocytes [19–21]. In line with the study's findings, guidelines recommend the daily use of HU by SCD patients to prevent painful episodes, vaso-occlusive crises, anemia, organ damage, and cerebrovascular complications such as stroke [20, 22, 23]. The latter has made HU the best alternative option for managing SCD patients, particularly in LMICs where health systems are immature and important rescue medicines and consumables such as nonsteroidal anti-inflammatory drugs (NSAIDs), opioids, and blood for transfusions are limited and costly [24, 25].

Moreover, the current study demonstrated that 75% of the study participants were on a 3500 mg or a higher dose of HU per week for more than 6 months. The HU dosing adopted in the low-middle income countries is the low-dose fixed dosing strategy compared to the maximal tolerated dosing strategy employed in the developed countries due to a lack of safety monitoring techniques. The approach could be a reason why some patients fail to reach the intended therapeutic concentration, while others get toxicity. The findings are consistent with what was reported in the phase III clinical trial conducted in Nigeria which reported that 10 mg/kg per day–20 mg/kg per day dose of HU for a median follow-up duration of 1.6 years to be efficacious and associated with minimal side effects for the prevention of secondary strokes and vaso-occlusive crisis among SCD patients [24]. The findings suggest that if HU therapy is well used in the recommended therapeutic regimen, it can prevent silent stroke, vaso-occlusive crisis, and other SCD complications, hence improving the quality of life of these patients [22, 26]. Nevertheless, more studies to assess the minimum and maximum efficacious HU doses are warranted in SCD-endemic regions.

Nevertheless, the study found that 3 patients experienced stroke pre- and during HU therapy, and 3 patients developed stroke after starting to use HU. Similar results were reported by Oniyangi et al., in which 5 SCD patients suffered a stroke after being placed on HU therapy [18]. Several factors are associated with the poor performance of HU including poor medication adherence, uses of incorrect doses, duration of HU uses, age, and comorbidities [17, 27, 28]. In addition, the recurrence of stroke episodes after using HU among SCD patients could be attributed to the occurrence of polymorphisms in the genes associated with its pharmacological effects and pharmacokinetics parameters. Besides, SCD is among the diseases associated with a significant number of polymorphisms. Recent studies recommend the importance of understanding the pharmacogenomics of various diseases to enable appropriate treatment of patients.

Lastly, the study found an even distribution of occurrence of stroke between male and female SCD patients. The findings are similar to what was found in Uganda in which the prevalence of stroke between male and female SCD patients was reported to be 48.2% and 51.9%, respectively [17]. Since both studies reported the indifferences as secondary outcomes, further work should be performed to assess if there is a difference in stroke prevalence between female and male SCD patients. However, the reported findings could be supported by the fact that SCD is a monogenic hereditary disorder. Also, in line with what was reported in Uganda (45%) and Nigeria (75%), most (46.4%) of the observed HU stroke occurred in SCD patients aged 6–12 years [17, 20]. The age group represents children who need caregivers' attention to adhere to treatment requirements including medications but also must attend school. Therefore, the observed high prevalence among SCD children aged 6–12 years could be attributed to poor adherence to treatment.

## 5. Limitation

Although the study thoroughly describes the prevalence of stroke pre- and during HU use among SCD from three RRHs, being a descriptive cross-sectional study, it fails to provide any strong statistical association between the demographic factors and the occurrence of stroke among participants. Also, it fails to provide a strong difference between the intervention and the control groups. The latter is true because stroke difference during HU use has different timeframes and cross-sectional design does not account for time effect. In addition, we missed some of the information such as body weight during initiation which is the key when describing HU dosing because some data were collected retrospectively.

## 6. Conclusion

The study showed that HU use is associated with a lower prevalence of new strokes. The findings suggest the need for stakeholders to institute measures to ensure eligible SCD patients are kept on HU. Besides, to ascertain the difference in the effect of HU in the prevention of stroke, we recommend cohort studies or randomized clinical trials.

## Figures and Tables

**Table 1:** Demographic characteristics of the study participants (*N* = 228).

Variables	*n* (%)
*Age category (years)*	
0–5	56 (24.6)
6–12	109 (47.8)
>12	63 (27.6)
*Sex*	
Female	124 (54.4)
Male	104 (45.6)
*Education level*	
Primary	181 (79.4)
Secondary	37 (16.2)
Tertiary	10 (4.4)
*Occupation (n* *=* *220)*	
Unemployed	209 (95.0)
Self-employed	8 (3.6)
Employed	3 (1.4)
*Marital status*	
Unmarried	226 (99.1)
Married	2 (0.9)
*Age at diagnosis of SCD category*	
0–5	172 (75.4)
6–12	47 (20.6)
>12	9 (3.9)

**Table 2:** Prevalence of stroke pre- and post-HU use among participants (*n* = 228).

Variables	*n* (%)
*Stroke status pre-hydroxyurea use*	
Yes	28 (12.3)
No	200 (87.7)
*Stroke status post-hydroxyurea use*	
Yes	6 (2.6)
No	222 (97.4)
Range of hydroxyurea used per week in mg (minimum-maximum) 6000 (1000–7000)	
*Dosage of HU used per week (mg)*	
<3500	59 (25.9)
3500	132 (57.9)
>3500	37 (16.2)
*Duration of HU use*	
< 2 years	132 (57.9)
≥ 2 years	96 (42.1)

**Table 3:** Pattern of stroke pre- and during HU use among SCD patients in relation to their demographic characteristics.

Variables	Stroke pre-HU use *n* (%)		Stroke during HU use *n* (%)		Stroke pre- and during HU use *n* (%)	
	No	Yes	No	Yes	No	Yes
*Age category (years), n* *=* *228*						
0–5	53 (94.6)	3 (5.4)	55 (98.2)	1 (1.8)	56 (100)	0 (0.0)
6–12	96 (88.1)	13 (11.9)	108 (99.1)	1 (0.9)	109 (100)	0 (0.0)
>12	51 (81.0)	12 (19.0)	59 (93.7)	4 (6.3)	60 (95.2)	3 (4.8)
*Sex, n* *=* *228*						
Female	110 (88.7)	14 (11.3)	121 (97.6)	3 (2.4)	123 (99.2)	1 (0.8)
Male	90 (86.5)	14 (13.5)	101 (97.1)	3 (2.9)	102 (98.1)	2 (1.9)
*Education level (n* *=* *228)*						
Primary	158 (87.3)	23 (12.3)	176 (97.2)	5 (2.8)	178 (98.3)	3 (1.7)
Secondary	33 (89.2)	4 (10.8)	36 (97.3)	1 (2.7)	37 (100)	0 (0.0)
Tertiary	9 (90.0)	1 (0.0)	10 (100)	0 (0.0)	10 (100)	0 (0.0)
*Occupation (n* *=* *220)*						
Unemployed	186 (89.0)	23 (11.0)	204 (97.6)	5 (2.4)	207 (99.0)	2 (1.0)
Self-employed	6 (75.0)	2 (25.0)	8 (100)	0 (0.0)	8 (100)	0 (0.0)
Employed	2 (66.7)	1 (33.3)	3 (100)	0 (0.0)	3 (100)	0 (0.0)
*Age at diagnosis category (n* *=* *228)*						
0–5	151 (87.8)	21 (12.2)	166 (96.5)	6 (3.5)	169 (98.3)	3 (1.7)
6–12	40 (85.1)	7 (14.9)	47 (100)	0 (0.0)	47 (100)	0 (0.0)
>12	9 (100)	0 (0.0)	9 (100)	0 (0.0)	9 (100)	0 (0.0)
*Dosage of HU used per week (mg)*						
<3500	56 (93.3)	4 (6.7)	59 (98.3)	1 (1.7)	60 (100.0)	0 (0.0)
3500	113 (86.3)	18 (13.7)	127 (96.9)	4 (3.1)	128 (97.7)	3 (2.3)
>3500	31(83.8)	6 (16.2)	36 (97.3)	1 (2.7)	37 (100.0)	0 (0.0)

## Data Availability

The data used to support the findings of this study are available from the corresponding author upon request.

## Abbreviations

HbF: Fetal hemoglobin
HbS: Sickle hemoglobin
HSCT: Hematopoietic stem cell transplantation
HU: Hydroxyurea
SCD: Sickle cell disease
LMICs: Low-middle-income countries
MUHAS: Muhimbili University of Health and Allied Sciences
RBCs: Red blood cells
RRH: Regional referral hospital
SCA: Sickle cell anemia
SPARCO: Sickle Pan-African Research Consortium
SPSS: Statistical Package for the Social Sciences
SSA: Sub-Saharan Africa
WHO: World Health Organization.
